# Isolation of detergent resistant microdomains from cultured neurons: detergent dependent alterations in protein composition

**DOI:** 10.1186/1471-2202-11-120

**Published:** 2010-09-22

**Authors:** Ritchie Williamson, Andrew J Thompson, Mika Abu, Abdul Hye, Alessia Usardi, Steven Lynham, Brian H Anderton, Diane P Hanger

**Affiliations:** 1 MRC Centre for Neurodegeneration Research, Institute of Psychiatry, King's College London, London, UK; 2 Biomedical Research Institute, University of Dundee, Ninewells Medical School, Dundee, UK

## Abstract

**Background:**

Membrane rafts are small highly dynamic sterol- and sphingolipid-enriched membrane domains that have received considerable attention due to their role in diverse cellular functions. More recently the involvement of membrane rafts in neuronal processes has been highlighted since these specialized membrane domains have been shown to be involved in synapse formation, neuronal polarity and neurodegeneration. Detergent resistance followed by gradient centrifugation is often used as first step in screening putative membrane raft components. Traditional methods of raft isolation employed the nonionic detergent Triton X100. However successful separation of raft from non-raft domains in cells is dependent on matching the detergent used for raft isolation to the specific tissue under investigation.

**Results:**

We report here the isolation of membrane rafts from primary neuronal culture using a panel of different detergents that gave rise to membrane fractions that differed in respect to cholesterol and protein content. In addition, proteomic profiling of neuronal membrane rafts isolated with different detergents, Triton X100 and CHAPSO, revealed heterogeneity in their protein content.

**Conclusions:**

These data demonstrate that appropriate selection of detergent for raft isolation is an important consideration for investigating raft protein composition of cultured neurons.

## Background

It is now accepted that lateral organisation occurs in membranes giving rise to distinct membrane domains characterised by differing lipid and protein composition. Membrane rafts are one such specialised membrane domain that has received considerable attention. Defined as small heterogeneous highly dynamic sterol- and sphingolipid-rich microdomains that can compartmentalise cellular processes [1], they have been reported to be integral to a wide range of cellular processes including cell signalling, endocytosis, and membrane trafficking [2]. Central to the study of membrane rafts is their detergent insolubility. Membrane rafts are enriched in cholesterol, sphingolipids and lipid modified proteins such as glycosylphosphatidyl (GPI)-anchored proteins. It is thought that the tight packing of sphingolipids long saturated acyl chains and intercalation of cholesterol allows for a more structured and rigid membrane organisation similar to the liquid ordered (Lo) phase of model membranes. This tight lipid packing separates them from the surrounding unsaturated gylcerolipid environment and also imparts resistance to detergent extraction [3].

Since the demonstration that GPI-anchored proteins were insoluble in non-ionic detergents including Triton X100 (Triton) at 4°C [4,5] and that this insolubility was cholesterol dependent [6], detergent resistant membrane (DRM) has subsequently become the operational definition for these isolated membrane domains. While detergent insolubility in itself is artifactual and does not accurately reflect pre-existing raft formation in cell membranes, detergent insolubility remains a powerful first step method for assigning potential membrane raft association.

Much of our current understanding of membrane raft biology has come from studies utilising the biochemical isolation of membrane rafts from epithelial and immune cells. More recently, the role of membrane rafts in central nervous system (CNS) function has been investigated and DRMs have been isolated from a variety of CNS tissue including both neuronal and glial cells as well as whole brain and synaptosomes [7-9]. Various functions have been attributed to these specialised membrane domains in neurons. Membrane rafts have been reported to be central to axonal growth cone guidance [10,11] as well as synapse formation and maintenance [12]. Receptor clustering has also been attributed to membrane rafts [13,14] including the recruitment of both excitatory AMPA and inhibitory GABA receptors to neuronal membrane raft domains [12,15]. Furthermore, accumulating evidence suggests a critical role for membrane rafts in neuronal signalling including endocytosis, trafficking, and neurotransmitter release (recently reviewed by Allen et al [16]). The role of membrane rafts in CNS function also extends to the pathogenesis of neurodegenerative disease including Alzheimer's disease (AD), prion disease and Parkinson's disease [16,17].

Since the postulation of the 'raft hypothesis' [18] and the isolation of membrane rafts as DRMs, a number of different detergents have been employed in the technical preparation of DRMs. The non-ionic detergent Triton is generally regarded as the gold-standard detergent for DRM preparation and has been used in initial studies characterising neuronal culture DRM composition [19,20]. Comparison of DRMs isolated with different detergents revealed altered protein and lipid composition [21-23] giving support to the concept of compositional heterogeneity underlying the functional heterogeneity of membrane rafts [24]. In addition, cell-dependent variation of DRM composition has also been reported [25] highlighting the importance of matching an appropriate detergent to specific tissue under investigation.

A growing body of literature supports an important role for membrane rafts in neuronal functioning. Given the observation that DRM compositional differences are cell type dependent we characterised DRMs isolated from primary neuronal cell culture using a panel of different detergents. The efficiency of DRM isolation was assessed by comparing the efficiency of recovery of cholesterol, total protein, and raft marker proteins in the isolated DRMs with total lysate. We further specifically characterised the proteomic composition of neuronal DRMs isolated with Triton, the gold-standard detergent for membrane raft isolation, and 3-(3-cholamindopropyl)-dimethylammonio-2-hydroxy-1-propanesulfonate (CHAPSO), the detergent that showed the best separation of DRMs from bulk lysate as determined by cholesterol and flotillin-1 content, by proteomic profiling using 1 D SDS-PAGE separation followed by LC-MS/MS (GeLC-MS/MS). We report here that DRMs can be isolated from primary neuronal cultures with a number of different detergents and that the composition of DRMs with respect to cholesterol and protein content is dependent on the specific detergent used. Furthermore, protein profiling using mass spectrometry and biochemical characterisation of neuronal DRMs isolated using different detergents lends support to the concept of raft heterogeneity.

## Results

### Isolation of neuronal DRMs using Triton

**Figure 1:**
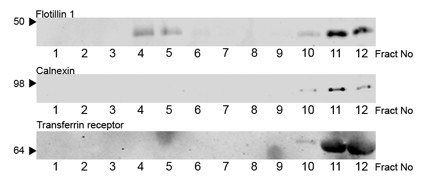
**Distribution of raft and non-raft components in Triton isolated neuronal DRMs**. Triton lysates of primary cortical cultures were fractionated by sucrose gradient ultracentrifugation. Equal volumes of each fraction was analysed by Western blotting with antibodies to the indicted proteins. Apparent molecular mass is indicated to the left.

Detergent resistant membrane rafts were isolated from primary cortical cells using extraction with the non-ionic detergent, Triton, followed by sucrose density centrifugation. Raft localisation was detected by SDS-PAGE of sucrose gradient fractions followed by Western blotting for the established raft protein flotillin-1. Figure 1 shows a typical distribution of flotillin-1 in fractions from the sucrose gradient. As expected, DRMs were found in the low-density fractions (fractions 4 and 5) corresponding to the 5% and 35% sucrose interface. A large proportion of flotillin was also detected in the high-density fractions (fractions 10-12). To examine the specificity of the DRM containing fractions and to ensure that traditional non-raft domains were fully solublised by Triton, Western blots of sucrose gradient fractions were analysed for the presence of the non-raft proteins calnexin and the transferrin receptor. Figure 1 illustrates a typical profile of calnexin and transferrin receptor immunolabelling of sucrose gradient fractions. Both calnexin and transferrin receptor were absent from the low-density DRM fractions 4 and 5 and concentrated in the high-density non-DRM fractions 10-12 demonstrating a clear separation between raft and non-raft proteins.

### Detergent specific distribution of flotillin-1 in sucrose gradients

**Figure 2:**
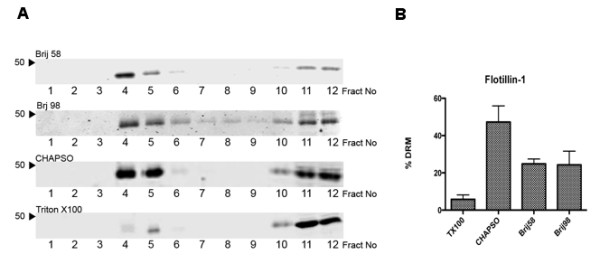
**Altered distribution of flotillin-1 in neuronal DRMs isolated with different detergents**. **A**. Equal volumes of primary cortical lysates were incubated with the indicated detergents at a final concentration of 1% (w/v) prior to fractionation by sucrose gradient centrifugation. Equal volumes of each fraction was analysed by Western blotting with an antibody to flotillin-1. Apparent molecular mass is indicated to the left. All blots are representative of at least three independent experiments. **B**. Quantification of flotillin-1 in neuronal DRM fractions isolated with indicated detergents expressed as % DRM enrichment of total gradient flotillin-1; *n *= 3.

It has been reported that isolation of DRMs with different detergents in differing cell populations gives rise to altered protein composition supportive of the idea of raft heterogeneity. To examine the effect of different detergents on identically cultured neuronal cells, we isolated DRMs with a panel of different detergents. Non-ionic Brij detergents have previously been employed to isolate lipid rafts from a variety of cells and tissue. In addition both the zwitterionic detergents CHAPS and CHAPSO have been used to isolate lipid rafts all of which have been reported to give rise to different compositions of raft-associated proteins in relation to Triton. We therefore examined the effect of the detergents, Triton, Brij 58, Brij 98, and CHAPSO, on the distribution of the lipid raft marker flotillin 1. We included Brij 98 in our study as this detergent has previously been employed to isolate DRMs at 37°C [25,26]. After sucrose gradient centrifugation a light scattering band was detected at the 5-35% sucrose interface for Triton, CHAPSO, Brij 58, and Brij 98. Figure 2 shows the distribution across the sucrose gradient fractions of the raft marker flotillin-1 for all detergents. For all detergents, flotillin-1 appeared to partition into two distinct sets of fractions, the low-density fractions (fractions 4 and 5) and the high-density fractions (fractions 10-12). However, there was a clear difference in the selectivity of detergents to enrich flotillin-1 in the low-density fractions. As noted previously, the most striking difference was observed in the distribution of flotillin-1 in the Triton extract. Here, the majority of flotillin-1 was found in the high-density soluble fractions and that only a minor fraction of total flotillin-1 partitioned into the low-density DRM fractions. Quantitation of Western blots revealed that DRM isolation by Triton extraction resulted in the lowest enrichment of flotillin-1, a mean ± SE of 7.5 ± 2.4% total flotillin-1 (Figure 2B) when compared to other detergents. In contrast, isolation of DRMs with the other detergents resulted in a greater enrichment of flotillin-1, CHAPSO (47.3 ± 8.6), Brij 58 (33.4 ± 8.8), and Brij 98 (25.3 ± 5.3). High intensity scanning of all the membranes revealed a clear separation of flotillin-1 positive bands between the high-density and low-density fractions and no presence of flotillin-1 was found in fractions 7-9. DRMs isolated with the different detergents were further examined for the presence of non-raft proteins. The transferrin receptor or calnexin could not be detected in the low-density fractions of any of the sucrose gradients isolated with any of the detergents and were detected solely in the high-density fractions (data not shown).

### Distribution of protein and cholesterol in sucrose gradient fractions from different detergents

**Figure 3:**
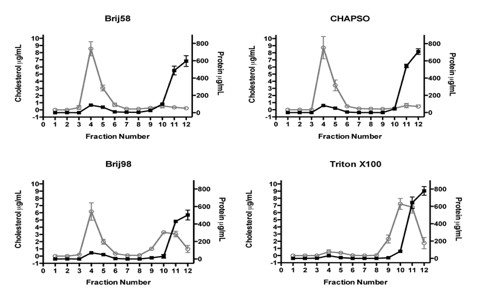
**Distribution of cholesterol and protein in sucrose gradient fractions of neuronal DRMs isolated with different detergents**. Equal volumes of primary cortical lysates were incubated with the indicated detergents at a final concentration of 1% (w/v) prior to fractionation by sucrose gradient centrifugation. Each 1 ml fraction was assayed for total protein (black lines) and cholesterol (grey lines). Protein and cholesterol content are expressed as μg/ml; *n *= 3.

Next we analysed the distribution of cholesterol and protein in the sucrose gradient fractions isolated with our panel of different detergents (Figure 3). The general pattern of protein distribution in the gradient fractions was similar for all the detergents tested with the largest amount of protein detected in the high-density fractions (fractions 10-12) and a smaller amount of protein spread out over the lower-density fractions (fractions 3-5). There was a striking difference in the distribution of cholesterol in the gradient fractions between the different detergents. For Triton gradients, the distribution of cholesterol mirrored the distribution of protein with the majority of cholesterol being detected in the high-density fractions and only a small proportion of cholesterol being isolated in the low-density fractions. The cholesterol distribution in the CHAPSO and Brij 58 gradients showed that the bulk cholesterol was found predominantly in the low-density fractions with only a trace being detected in the high-density fractions. For the Brij 98 gradients there was a clear bimodal distribution of cholesterol into both the low-density fractions and the high-density fractions although the peak for cholesterol content was higher in the low-density fractions than for the high-density fractions, similar to the cholesterol distribution reported in other neuronal studies [27].

The total protein and total cholesterol content as well as % DRM enrichment from sucrose gradients fractions are summarised in Table 1. Triton gradients had the lowest % DRM cholesterol enrichment with a mean ± SE of 4.2 ± 1.7% while CHAPSO gradients had the highest % DRM enrichment of cholesterol, 84.0 ± 6.9%.

**Table 1:** DRM enrichment of protein and cholesterol

Detergent	Total gradient cholesterol (μg)	% DRM cholesterol enrichment	Total gradient protein (mg)	% DRM protein enrichment
Triton X100	19.0 ± 2.9	4.2 ± 1.7	1.55 ± 0.109	2.8 ± 0.68
CHAPSO	14.3 ± 2.2	84.0 ± 6.9	1.43 ± 0.058	9.3 ± 0.49
Brij58	14.3 ± 1.7	81.2 ± 5.6	1.38 ± 0.130	10.9 ± 0.33
Brij98	17.3 ± 2.2	46.9 ± 5.7	1.10 ± 0.048	10.9 ± 0.32

**Figure 4:**
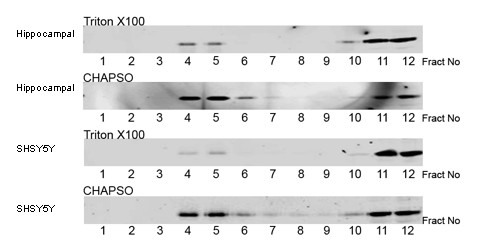
**Distribution of flotillin-1 in DRMs isolated from hippocampal neurons and SHSY5Y cells**. Equal volumes of primary hippocampal lysates or SHSY5Y lysates were incubated with the indicated detergents at a final concentration of 1% (w/v) prior to fractionation by sucrose gradient centrifugation. Equal volumes of each fraction was analysed by Western blotting with an antibody to flotillin-1. Apparent molecular mass is indicated to the left.

As CHAPSO gradients yielded the highest distribution of the raft marker flotillin-1 and cholesterol in the low density fractions and Triton yielded the lowest distribution of flotillin-1 and cholesterol in the low-density fractions we next investigated the distribution of this marker in other primary hippocampal cultures and the neuroblastoma cell line SHSY5Y (Figure 4). Similar to primary cortical cultures, DRMs isolated with CHAPSO displayed an enrichment of flotillin-1 in the low-density fractions from both hippocampal and SHSY5Y cells when compared to DRMs isolated with Triton.

### Proteomic profile of neuronal DRMs

**Figure 5:**
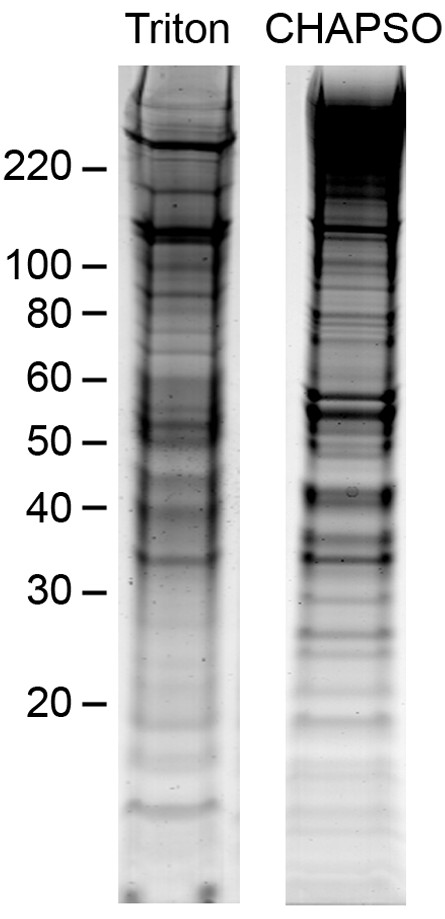
**Colloidal Coomassie profile of DRMS isolated from hippocampal neurons**. Concentrated DRMs isolated from hippocampal neurones were resolved by SDS-PAGE followed by colloidal Coomassie staining. Apparent molecular mass is indicated to the left.

The protein composition of DRMs isolated with the two detergents Triton and CHAPSO were comprehensively characterised by proteomic profiling. Isolated hippocampal DRM fractions were collected and concentrated by ultra-centrifugation. Whole DRM preparations were resolved by SDS-PAGE, visualised by staining with colloidal Coomassie blue, and the entire gel lane excised for in-gel digestion and LC-MS/MS analysis (Figure 5). In total, 2583 and 1876 unique peptide sequences were confidently assigned in the CHAPSO and Triton raft preparations, respectively. This corresponded to the identification of 437 and 288 proteins in the two preparations, consistent with the higher protein yield observed for CHAPSO DRM isolation. Additional file 1, Table S1 gives the complete list of proteins identified in each preparation along with Swissprot Entry numbers and proteomic characterisation. Many proteins commonly isolated in DRM preparations were identified in both profiles, 234 proteins where common to both DRMs while CHAPSO raft preparations had 203 unique proteins and Triton raft preparations had 54 unique proteins. Table 2 gives a summary of proteins identified in both the CHAPSO and Triton raft preparations. Proteins common to both preparations included tubulin, actin, and associated structural proteins; anion-selective and calcium channel/transporter proteins; GTP-binding proteins, ras-family members and other cell signalling proteins; and the raft marker proteins flotillin-1, flotillin-2 and Thy-1. Similarly to other neuronal DRM preparations, numerous proteins were identified that are not normally found in non-neuronal DRMs. These included the neuronal structural proteins brain acid soluble protein, neuronal growth regulator 1 and several contactins; and various synaptic proteins including syntaxins, neuroligins and neuromodulin.

**Table 2:** The number of proteins identified in the CHAPSO and Triton raft preparations according to cellular component categories

Cellular component	Unique protein assignments		
	
	CHAPSO	TX100	Combined
Cytoplasm	31	25	33
Cytoskeleton/Filament	24	42	43
Endosome/Lysosome	14	4	14
ER/Golgi	54	9	54
Mitochondrion	69	54	77
Nucleus	6	5	7
Other	80	45	91
Plasma membrane	104	62	106
Synapse	45	35	53
Vesicle	10	7	11
Total Proteins	437	288	489

**Figure 6:**
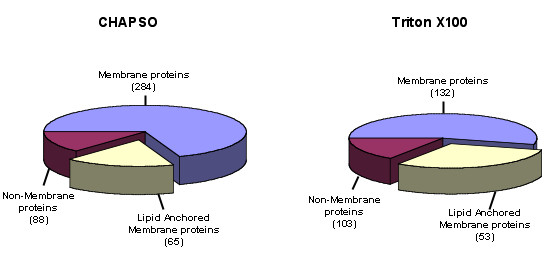
**Distributions of membrane and non-membrane proteins identified by proteomic profiling of the CHAPSO and Triton DRM preparations**. Membrane localisations were assigned based on protein annotations listed in the Uniprot database in conjunction with the transmembrane prediction algorithm Phobius. The pie slice areas represent the number of proteins in each category (listed in parentheses) as a proportion of the total number of proteins identified in each DRM preparation.

Annotation of the protein profiles revealed proteomic differences between the two DRM preparations. In the CHAPSO preparation, 349 of 437 (80%) of the proteins identified were classified as membrane proteins, compared to 185 of 288 (64%) for Triton (Figure 6). Of these, 65 of the membrane proteins from the CHAPSO preparation were known to incorporate lipid modifications, compared to 53 in the Triton preparation. Of interest, slightly more non-membrane proteins were identified in the Triton preparation compared to the CHAPSO preparation (103 vs 88 proteins, respectively). Comparison of cellular compartmentalisation of the proteins isolated in each of the preparations revealed an increase in the relative amount (expressed as percent of total DRM proteins) of cytoskeletal/filament proteins isolated in the Triton preparation compared to the CHAPSO preparation (Table 2). There was also an enrichment of endoplasmic reticulum (ER) and Golgi associated proteins in the CHAPSO preparation. Both preparations had a similar proportion of plasma membrane associated proteins suggesting that the enrichment of membrane proteins in the CHAPSO preparation was in part due to the increased isolation of membrane bound ER, Golgi, and endosome associated proteins. In addition, both preparations contained an identical enrichment of GPI-anchored proteins, although more lipid-anchored proteins were isolated in the CHAPSO preparation. Collectively, this data indicated a higher and more specific recovery of membrane and lipid anchored proteins in the CHAPSO DRM preparations. Proteins were further characterised with respect to function. Overall, there appeared to be a similar profile of proportional enrichment by functional classification with one obvious exception. There was a greater than two-fold increase in cell structure and adhesion proteins in the Triton preparation.

To investigate further proteomic differences, the relative abundance of proteins identified in the two samples was estimated by crude semi-quantitative analysis using spectral counting (Additional file 1, Table S1), similar to recent proteomic studies [28,29]. Several proteins including the well-characterised raft marker proteins flotillin-1 and -2 and Thy-1 were identified by a high number of peptides in both preparations, suggesting they were abundantly present in both raft preparations. In contrast, numerous other proteins were evidenced by confident detection of many peptides in one sample but markedly fewer or no peptides in the other, clearly indicating differing enrichment of some specific protein sub-classes. This included clear enrichment of some channels/receptors, synaptic proteins and rab proteins by CHAPSO but not by Triton (Additional file 2, Table S2), and conversely enrichment of several cell adhesion/structural components and ras GTPase activators/regulators by Triton but not by CHAPSO (Additional file 3, Table S3).

## Discussion

We report here detergent-dependent variation in the enrichment of isolated DRMs with respect to cholesterol and protein. In addition, enrichment of the lipid raft marker flotillin-1 in the isolated DRMs differed greatly between the detergents tested. Of note, Triton solubilised the majority of flotillin-1 in all cells tested (hippocampal, cortical, SHSY5Y) with less than 10% of total flotillin-1 partitioning into low-density fractions. The order of DRM enrichment for flotillin-1 is CHAPSO > Brij 58 > Brij 98 > Triton. There was little difference in the amount of protein recovered in the low-density fractions between CHAPSO and both the Brij detergents. However, consistent with the poor enrichment of flotillin-1 in the Triton extracted DRMs, Triton proved to be very poor at enriching protein in low-density fractions when compared with the other detergents. Membrane rafts by definition are cholesterol rich, and so we examined the ability of each of the detergents to enrich for cholesterol in DRMs. In primary neuronal cultures, the order of DRM enrichment for cholesterol is CHAPSO > Brij 58 > Brij 98 > Triton, identical to DRM enrichment for flotillin 1. This order of selectivity of detergents in their ability to enrich DRMs is cell type-dependent as both CHAPSO and Triton have been reported to be equally effective at DRM enrichment and more effective than both Brij 98 and Brij 58 in Jurkat and Madin-Darby canine kidney cells [25]. In addition, we show here that membrane rafts can be isolated from neuronal cells at physiological temperatures with Brij 98 and that these DRMs are particularly enriched in cholesterol and flotillin-1. Our comparison of different detergents clearly showed the greatest difference between CHAPSO and Triton in their ability to enrich cholesterol, protein, and known membrane raft markers in DRMs.

Proteomic characterisation of neuronal DRMs isolated with CHAPSO and Triton further demonstrated considerable differences between the two DRMs. As expected, there was a high representation of plasma membrane proteins within the isolated DRMs. Consistent with other non-neuronal proteomic studies of DRMs, neuronal DRMs contained multiple proteins from a number of intracellular organelles [30,31]. There was a high concentration of mitochondrial-associated proteins present in CHAPSO and Triton DRMs, 16% and 19% respectively. As yet there is no consensus about the presence of mitochondrial proteins as membrane raft components. Numerous proteomic studies have reported the presence of mitochondrial proteins [32,33], in particular ATP synthase subunits. However, only a few particular mitochondrial proteins are not restricted to mitochondria and are resident proteins in membrane rafts [30]. By determining the cholesterol dependence of mitochondrial proteins and application of high resolution linear density centrifugation, it has been reported that the majority of these proteins co-purify with DRMs but are not raft specific [34]. Our results also suggest that mitochondrial proteins isolated in DRMs are not cholesterol dependent as there was a 15-fold increase in the cholesterol extracted DRMs compared to Triton extracted DRMs but a similar representation of mitochondrial proteins in both preparations. Our profile also included a number of ER Golgi and endosomal proteins consistent with recent studies identifying DRMs in these intracellular organelles [35-40]. In addition there was an enrichment of cell type specific neuronal proteins such as synaptic and adhesion proteins. Membrane rafts are reported to be involved in a number of different cellular processes including cell signalling and endocytosis. The abundance of kinases and phosphatases as well as ras GTPases detected in our DRMs is in support of these domains having such roles. In addition, the neuronal DRMs characterised here also contained an abundance of synaptic and receptor proteins specific to neurotransmission and synaptic maintenance.

Assigning functional classification and cellular compartmentalisation highlights a number of proteins that should not be present in membrane rafts but are present in both DRM preparations, such as the non-membrane nuclear histones. While these proteins clearly seem out of place in a profile of putative membrane raft proteins, other proteins which would appear to be contaminants may indeed be membrane raft resident. The transferrin receptor protein, which is traditionally regarded as a non-raft marker was identified in CHAPSO isolated DRMs by mass spectrometry. As suggested by other DRM proteomic studies this could also be a contaminant, however we cannot exclude the possibility that mass spectrometry has identified the small proportion of palmitoylated transferrin receptor that has been reported to translocate into membrane raft domains [41]. Recent proteomic studies have suggested that approximately one-third of proteins isolated by detergent extraction are either contaminants or co purifying non raft associated proteins [42]. Further work utilising cholesterol depletion studies will aid in delineating membrane raft and membrane raft-associated proteins from protein contaminants.

Our proteomic data show that there is considerable overlap between the two DRM preparations. Approximately 81% of proteins identified in the Triton DRM were also detected in the CHAPSO DRM. This included all the GPI-linked proteins and all the dually acylated (palmitoylated and myristoylated) proteins, lipid modifications involved in targeting proteins to membrane rafts [43]. The data would suggest that both detergents isolate a core set of membrane raft proteins, plus a detergent specific enrichment of a subset of proteins. Detergent extraction also disrupts lipid protein interactions, and the extent of disruption of these lipid protein interactions is evident in both DRMs. The cortical actin cytoskeleton has been reported to be involved in membrane raft formation. The enrichment of cytoskeletal proteins in the Triton DRM preparation suggests that Triton is particularly poor at disrupting the membrane raft cytoskeleton interaction, while CHAPSO is more stringent at disrupting actin membrane raft interactions.

Over 150 more proteins were identified in the DRM preparation isolated using CHAPSO compared to Triton X100 providing a more comprehensive overview of the DRM proteomic composition in keeping with the increase in protein isolated in the respective DRMs. In addition, there was a clear increase in selectivity of CHAPSO in enriching both ER/Golgi and endosomal proteins when compared to Triton.

Importantly, some known raft proteins including cytochrome B5 reductase, which clusters in neuronal plasma membrane rafts [44] and plasma membrane Ca^2+ ^-ATPases [27] were only identified in the CHAPSO DRM preparation. Critically for neurological studies, receptors and channels instrumentally involved with synaptic neurotransmission including glutamate/aspartate excitatory amino acid transporter-1 and -2, glutamate receptors AMPA-1 and -2, and GABA receptors, which are known to cluster in membrane rafts [16], were greatly enriched in the CHAPSO DRM preparation. Similarly, proteins involved in neurological disorders including γ-secretase components, integral to processing of the amyloid precursor protein implicated in the progression of Alzheimer's disease [45], neurexins and neuroligins implicated in autism and schizophrenia [46-48] were also clearly enriched (Table 3).

**Table 3:** Selected known raft proteins and proteins associated with neurological disease identified in the CHAPSO and Triton preparations

	Accession	Protein Description	# Assigned spectra	
			
			CHAPSO	TX100
Known raft proteins	FLOT1_RAT	Flotillin-1	30	41
	FLOT2_RAT	Flotillin-2	23	33
	NB5R1_RAT	NADH-cytochrome b5 reductase 1	4	
	NB5R3_RAT	NADH-cytochrome b5 reductase 3	13	
	THY1_RAT	Thy-1 membrane glycoprotein	6	11
				
				
Neurological disease	BASI_RAT	Basigin	6	2
	PRIO_RAT	Major prion protein	6	6
	NRX1A_RAT	Neurexin-1-alpha	2	
	NRX2A_RAT	Neurexin-2-alpha	3	
	NRX3A_RAT	Neurexin-3-alpha	17	19
	NLGN1_RAT	Neuroligin-1	3	1
	NLGN3_RAT	Neuroligin-3	8	2
	NICA_RAT	Nicastrin	18	
	PSN1_RAT	Presenilin-1	4	
	TMEDA_RAT	Transmembrane emp24 domain-containing protein 10	9	

## Conclusions

These results indicate that CHAPSO DRM preparations are especially suited to directly investigate the role of membrane rafts on neurotransmission and synaptic dysfunction in neuronal culture. The observation that greater than 95% of cholesterol was not recovered in the DRMs from Triton extracted cells strongly indicates that Triton is highly stringent solubiliser of neuronal cells and is not particularly suited to the investigation of membrane rafts in primary neuronal cells. However, without the functional specificity of cholesterol depletion we cannot comment on whether CHAPSO or Triton is more or less specific in isolating raft proteins.

The study presented here provides the first proteomic characterisation of cultured neuronal membrane rafts and highlights the inherent limitations of using a single step extraction protocol to isolate DRMs. Nonetheless, detergent based studies on membrane rafts are a useful first step protocol for examining putative membrane raft association and in comparative studies.

## Methods

### Reagents

All chemicals unless otherwise stated were purchased from Sigma (Gillingham, UK). The following primary antibodies were used against: fyn, flotillin-1 (BD Transduction Laboratories, Lexington, KY, USA); phosphotyrosine (4G10; Upstate, Lake Placid, NY, USA); actin (Abcam, Cambridge, UK).

### Primary neuronal cultures

Primary cortical cultures were prepared from day 18 rat embryos as previously described [49]. Primary cortical cells were plated onto poly-L-lysine (10 μg/ml) coated 10 cm dishes at a 7 × 10^6 ^cells/dish. Primary hippocampal cells were plated onto poly-L-lysine coated 10 cm dishes at 3 × 10^6 ^cells/dish. Cultures were maintained in Neurobasal medium containing B27 supplement, 2 mM glutamine and 20 μg/ml gentamicin solution. Primary cortical cells were cultured for 7 days and primary hippocampal cells were cultured for 14-21 days before being used for the membrane raft isolation. Under these conditions, cultures were almost exclusively neuronal as determined by routine staining with antibodies to neurofilaments and glial fibrillary acidic protein. All studies were performed under regulations permitted by UK Home Office.

SHSY5Y cultures were maintained as previously described [50]. Cells were cultured in Eagles minimum essential medium (EMEM-F12 1:1) supplemented with 15% v/v FCS, 2 mM glutamine and 20 μg/ml gentamicin soulution.

### Detergents

Detergents used were Brij 58, Brij 98, Triton X100 and CHAPSO.

### Detergent resistant membrane raft isolation

All procedures unless stated otherwise were carried out on ice and all buffers were kept at 0-4°C for the duration of the procedure. For whole cell lipid raft isolation 2 × 10 cm dishes (7 × 10^6 ^cells/dish) were lysed directly in 1 ml MBS buffer (25 mM MES, 150 mM NaCl, pH 6.5) containing 10 mM MgCl_2_, 10 mM NaF, 2 mM Na_3_VO_4_, 1 mM EGTA, 5 mM DTT, 0.2 mM PMSF. 1 ml of lysate were then added to 1 ml of MBS containing either 2% w/v Brij 58, 2% w/v Brij 98, 2% w/v Triton X100, 2% w/v CHAPSO, to yield a final detergent concentration of 1% w/v for all detergents. Lysates were incubated on ice for 30 min followed by Dounce homogenisation (18 strokes). In the case of Brij 98, the lysate was incubated at 37°C for 10 min prior to Dounce homogenisation. The homogenate (1 ml) was then mixed with 1 ml of 90% (w/v) sucrose in MBS buffer and placed in a 12 ml ultracentrifuge tube. A discontinuous 5-35-45% sucrose gradient was formed by layering 4 ml of 35% (w/v) sucrose in MBS solution on top of the 2 ml homogenate, followed by 4 ml 5% (w/v) sucrose in MBS solution. The sample was then centrifuged at 39,000 rpm for 18 h at 4°C in a Beckman SW41 rotor. A light scattering band at the 5-35% interface was identified that was enriched in flotillin, indicating the presence of membrane rafts. 12 × 1 ml fractions were collected from the top of each gradient. To concentrate membrane rafts, fractions 4-5 were diluted in 10 ml MBS buffer containing 10 mM NaF, and 2 mM Na_3_VO_4 _and centrifugating at 39,000 rpm for 1 hr in a Beckman SW41 rotor. The membrane raft-containing pellet was solubilised in 100 μl 20 mM Tris/8 M urea pH 7.4 containing 10 mM NaF, 2 mM Na_3_VO_4_, 5 mM DTT, and 0.2 mM PMSF.

### Cholesterol and protein measurements

Immediately prior to analysis, all raft fractions were passed through a fine gauge needle (22 g) twice before aliquots were taken from each of the fractions for each of the following methods. Total protein in each fraction was determined using the BCA protein assay kit (Pierce). Bovine serum albumin diluted in different fractions isolated from sucrose gradient centrifugation of relevant detergent blanks was used as standard. Total cholesterol content in each fraction was measured fluorimetrically using the Amplex Red Cholesterol Assay Kit (Invitrogen, UK).

### Western Blotting

Proteins were resolved by SDS-PAGE using 10% (w/v) polyacrylamide. Proteins were transferred to nitrocellulose (Schleicher & Scheull, Dassel, Germany) and immunodetection was performed using Alexa Fluor^® ^conjugated goat anti-mouse or IRDye™ 800 conjugated affinity purified anti-Rabbit IgG (Rockland Immunochemicals Inc, Gilbertsville, PA, USA) in conjunction with an Odyssey Infrared Imaging System (Li-Cor Biosciences, Lincoln, NE, USA). Image analysis and quantification measurements were performed using the Odyssey Infrared Imaging System application software (Li-Cor Biosciences, Lincoln, NE, USA).

### GelC-MS/MS

Concentrated membrane rafts were solubilised in 4 × Laemmli sample buffer (Invitrogen) and membrane raft proteins resolved on a 4-12% Bis-Tris gel (Invitrogen). Protein bands were visualised by briefly staining with colloidal Coomassie blue and the gel lane excised into 30 sections for in-gel digestion according to established protocols [51]. Briefly, the proteins were reduced with 10 mM DTT in 100 mM ammonium bicarbonate and free cysteine residues were alkylated with 55 mM iodoacetamide in 100 mM ammonium bicarbonate. The proteins were then digested with trypsin (Roche Diagnostics, UK) for 2 h at 37°C then overnight at room temperature. In-gel digested samples were fractionated by reversed-phase chromatography using an Ultimate LC system (Dionex, Camberley, UK). Peptides were resolved on a C18 PepMap column (75 mm I.D.) using a three-step linear gradient of 0-48% acetonitrile/0.05% formic acid over 120 min at a flow rate of 200 nl/min. Peptides were ionized by electrospray ionization using a Z-spray source fitted to a QTof-micro (Waters Ltd, Elstree, UK) operating under Masslynx v3.5 software. The instrument was run in automated switching mode, selecting precursor ions based on their intensity and charge state for sequencing by collision-induced fragmentation. The MS/MS was performed using collision energy profiles that were chosen based on the mass/charge (m/z) ratios. Raw data were recalibrated against internal tryptic peptides and processed into peak lists using ProteinLynx Global Server V2.2.5 with the following MS/MS processing parameters: smoothing by Savitzky-Golay method, 2 iterations, 4 channels; peak centroiding top 80%, no deisotoping or background subtraction.

### Protein identification

Proteins were identified by searching the MS peak list data for each raft profile against the Swissprot database from Uniprot version 13.2 http://www.expasy.org/ as a single merged search using the Mascot V2.2 search engine http://www.matrixscience.com/. The following parameter specifications were employed: Precursor ion mass tolerance 1.2 Da, fragment ion mass tolerance 0.6 Da, species restricted to *rattus norvegicus *(6939 entries), tryptic peptides with up to two missed cleavages, variable modifications: carbamidomethylation of cysteine residues, methionine oxidation, and pyroglutamisation of N-terminal glutamine residues. A high ion mass tolerance was used to account for incorrect precursor isotope selection that occasionally occurs during data acquisition with Masslynx V3.5. With the exception of these cases, mass accuracy was typically less than 50 ppm. Scaffold V2.1.1 (Proteome Software Inc., Portland, OR) was used to validate MS/MS based peptide and protein identifications. DAT files for each raft profile were loaded into Scaffold as a combined MuDPIT experiment. Peptide identifications were accepted if they could be established at greater than 95% probability as specified by the Peptide Prophet algorithm [52]. Protein identifications were automatically accepted if they contained at least one unique peptide assignment and could be established at 99% identification probability or greater by the Protein Prophet algorithm [53]. Protein identifications scoring between 95% and 99% probability were validated by manual inspection according to accepted procedures. All other returned protein hits were rejected. Functional and localisation annotations for proteins were assigned using the information in the Uniprot database http://www.uniprot.org and the transmembrane prediction algorithm Phobius [54].

## Competing interests

The authors declare that they have no competing interests.

## Authors' contributions

RW conceived the study and carried out the biochemical separation of membrane rafts and Western blotting and protein concentration measurements. In addition RW participated in the design of the study and drafted the manuscript. AJT carried out the mass spectrometry and protein identification and participated in drafting the manuscript. MA performed the cholesterol measurements. AH participated in establishing and maintaining primary neuronal and cell line cultures. AU participated in the biochemical separation of membrane rafts, Western blotting and neuronal culture. SL participated in mass spectroscopy and protein identification. BHA contributed to manuscript preparation and study design. DPH contributed to manuscript preparation and study design. All authors read and approved the final manuscript.

## Supplementary Material

Additional file 1 **Supplementary Table S1**. Click here for file

Additional file 2 **Supplementary Table S2**. Click here for file

Additional file 3 **Supplementary Table S3**. Click here for file
