# Supplementary material for: Isolation of detergent resistant microdomains from cultured neurons: detergent dependent alterations in protein composition
Source: BMC Neurosci. 2010 Sep 22;11:120. doi: 10.1186/1471-2202-11-120 (PMC2955047; doi:10.1186/1471-2202-11-120)
Supplement: Additional file 2 — Supplementary Table S2. [file 1471-2202-11-120-S2.DOC]

|  | **Accession** | **Protein Description** | **# Assigned Spectra** | |
| --- | --- | --- | --- | --- |
|  |  |  | **CHAPSO** | **TX100** |
| **Ras-related proteins** | RB11A_RAT | Ras-related protein Rab-11A | 8 | 1 |
|  | RAB14_RAT | Ras-related protein Rab-14 | 9 | 1 |
|  | RAB18_RAT | Ras-related protein Rab-18 | 4 |  |
|  | RAB1A_RAT | Ras-related protein Rab-1A | 15 | 4 |
|  | RAB1B_RAT | Ras-related protein Rab-1B | 8 |  |
|  | RAB2A_RAT | Ras-related protein Rab-2A | 9 | 1 |
|  | RAB31_RAT | Ras-related protein Rab-31 | 4 |  |
|  | RAB35_RAT | Ras-related protein Rab-35 | 3 | 2 |
|  | RAB3A_RAT | Ras-related protein Rab-3A | 23 | 4 |
|  | RAB3C_RAT | Ras-related protein Rab-3C | 2 |  |
|  | RAB4B_RAT | Ras-related protein Rab-4B | 4 |  |
|  | RAB6A_RAT | Ras-related protein Rab-6A | 8 |  |
|  | RAB7A_RAT | Ras-related protein Rab-7a | 18 | 3 |
|  | RAB8A_RAT | Ras-related protein Rab-8A | 2 |  |
|  | RALA_RAT | Ras-related protein Ral-A | 2 | 1 |
|  | RAP1B_RAT | Ras-related protein Rap-1b | 16 | 6 |
|  | RAP2B_RAT | Ras-related protein Rap-2b | 9 | 5 |
|  |  |  |  |  |
| **Channels and Transporters** | SFXN1_RAT | Sideroflexin-1 | 15 | 1 |
|  | SFXN3_RAT | Sideroflexin-3 | 14 | 2 |
|  | SFXN5_RAT | Sideroflexin-5 | 7 |  |
|  | S4A7_RAT | Sodium bicarbonate cotransporter 3 | 5 |  |
|  | SCN2A_RAT | Sodium channel protein type 2 subunit alpha | 7 |  |
|  | NAC1_RAT | Sodium/calcium exchanger 1 | 35 |  |
|  | NAC2_RAT | Sodium/calcium exchanger 2 | 25 |  |
|  | AT1A1_RAT | Sodium/potassium-transporting ATPase subunit alpha-1 | 89 | 18 |
|  | AT1A2_RAT | Sodium/potassium-transporting ATPase subunit alpha-2 | 27 |  |
|  | AT1A3_RAT | Sodium/potassium-transporting ATPase subunit alpha-3 | 386 | 115 |
|  | AT1B1_RAT | Sodium/potassium-transporting ATPase subunit beta-1 | 50 | 16 |
|  | AT1B3_RAT | Sodium/potassium-transporting ATPase subunit beta-3 | 3 |  |
|  | S38A3_RAT | Sodium-coupled neutral amino acid transporter 3 | 2 |  |
|  | S4A10_RAT | Sodium-driven chloride bicarbonate exchanger | 6 |  |
|  | S12A5_RAT | Solute carrier family 12 member 5 | 23 |  |
|  | GTR3_RAT | Solute carrier family 2, facilitated glucose transporter member 3 | 13 |  |
|  | S23A2_RAT | Solute carrier family 23 member 2 | 2 |  |
|  | S4A4_RAT | Electrogenic sodium bicarbonate cotransporter 1 | 13 |  |
|  | S4A8_RAT | Electroneutral sodium bicarbonate exchanger 1 | 2 |  |
|  | LAT1_RAT | Large neutral amino acids transporter small subunit 1 | 16 |  |
|  | AT2B1_RAT | Plasma membrane calcium-transporting ATPase 1 | 96 |  |
|  | AT2B2_RAT | Plasma membrane calcium-transporting ATPase 2 | 27 |  |
|  | AT2B3_RAT | Plasma membrane calcium-transporting ATPase 3 | 9 |  |
|  | AT2B4_RAT | Plasma membrane calcium-transporting ATPase 4 | 3 |  |
|  | AT2A2_RAT | Sarcoplasmic/endoplasmic reticulum calcium ATPase 2 | 35 | 2 |
|  | 4F2_RAT | 4F2 cell-surface antigen heavy chain | 81 | 5 |
|  | ADT1_RAT | ADP/ATP translocase 1 | 178 | 23 |
|  | ADT2_RAT | ADP/ATP translocase 2 | 49 | 5 |
|  |  |  |  |  |
| **Synaptic Function** | EAA1_RAT | Excitatory amino acid transporter 1 | 85 | 8 |
|  | EAA2_RAT | Excitatory amino acid transporter 2 | 82 | 2 |
|  | GBRA2_RAT | Gamma-aminobutyric acid receptor subunit alpha-2 | 2 |  |
|  | GBRA5_RAT | Gamma-aminobutyric acid receptor subunit alpha-5 | 2 |  |
|  | GBRB3_RAT | Gamma-aminobutyric acid receptor subunit beta-3 | 4 |  |
|  | GABR1_RAT | Gamma-aminobutyric acid type B receptor subunit 1 | 13 | 1 |
|  | GABR2_RAT | Gamma-aminobutyric acid type B receptor subunit 2 | 7 | 1 |
|  | NMDZ1_RAT | Glutamate [NMDA] receptor subunit zeta-1 | 1 |  |
|  | GRIA1_RAT | Glutamate receptor 1 (AMPA1) | 12 | 1 |
|  | GRIA2_RAT | Glutamate receptor 2 (AMPA2) | 56 | 3 |
|  | GRM5_RAT | Metabotropic glutamate receptor 5 | 5 |  |
|  | SCAM1_RAT | Secretory carrier-associated membrane protein 1 | 8 |  |
|  | SCAM5_RAT | Secretory carrier-associated membrane protein 5 | 19 |  |
|  | SC6A1_RAT | Sodium- and chloride-dependent GABA transporter 1 | 23 | 1 |
|  | S6A11_RAT | Sodium- and chloride-dependent GABA transporter 3 | 12 |  |
|  | SYN1_RAT | Synapsin-1 | 1 | 1 |
|  | SYN2_RAT | Synapsin-2 |  | 8 |
|  | SV2A_RAT | Synaptic vesicle glycoprotein 2A | 35 |  |
|  | SV2B_RAT | Synaptic vesicle glycoprotein 2B | 6 |  |
|  | SNG1_RAT | Synaptogyrin-1 | 3 | 1 |
|  | SYPH_RAT | Synaptophysin | 44 | 11 |
|  | SNP25_RAT | Synaptosomal-associated protein 25 | 19 | 6 |
|  | SYT1_RAT | Synaptotagmin-1 | 58 | 6 |
|  | SYT12_RAT | Synaptotagmin-12 | 1 |  |
|  | SYT5_RAT | Synaptotagmin-5 | 1 |  |
|  | STX18_RAT | Syntaxin-18 | 4 |  |
|  | STX1A_RAT | Syntaxin-1A | 21 |  |
|  | STX1B_RAT | Syntaxin-1B | 31 |  |
|  | STX6_RAT | Syntaxin-6 | 5 |  |
|  | STX7_RAT | Syntaxin-7 | 10 | 1 |
|  | STX8_RAT | Syntaxin-8 | 4 |  |
|  | STXB1_RAT | Syntaxin-binding protein 1 | 4 | 5 |
|  | STXB5_RAT | Syntaxin-binding protein 5 | 6 |  |
|  | VAMP1_RAT | Vesicle-associated membrane protein 1 | 4 |  |
|  | VAMP2_RAT | Vesicle-associated membrane protein 2 | 47 | 8 |
|  | VAMP3_RAT | Vesicle-associated membrane protein 3 | 6 |  |
|  | VAPA_RAT | Vesicle-associated membrane protein-associated protein A | 16 | 3 |
|  | VAPB_RAT | Vesicle-associated membrane protein-associated protein B | 4 |  |
|  | NSF_RAT | Vesicle-fusing ATPase | 12 | 4 |
|  | SC22B_RAT | Vesicle-trafficking protein SEC22b | 13 |  |
|  | VIAAT_RAT | Vesicular inhibitory amino acid transporter | 5 |  |
|  |  |  |  |  |
| **Alzheimer Disease proteins** | BASI_RAT | Basigin | 6 | 2 |
|  | NICA_RAT | Nicastrin | 18 |  |
|  | TMEDA_RAT | Transmembrane emp24 domain-containing protein 10 | 9 |  |
|  | PSN1_RAT | Presenilin-1 | 4 |  |
|  |  |  |  |  |
|  |  |  |  |  |
|  |  |  |  |  |

Table 4. Selected protein categories enriched in CHAPSO raft preparations.
