# Supplementary material for: Isolation of detergent resistant microdomains from cultured neurons: detergent dependent alterations in protein composition
Source: BMC Neurosci. 2010 Sep 22;11:120. doi: 10.1186/1471-2202-11-120 (PMC2955047; doi:10.1186/1471-2202-11-120)
Supplement: Additional file 3 — Supplementary Table S3. [file 1471-2202-11-120-S3.DOC]

|  | **Accession** | **Protein Description** | **# Assigned Spectra** | |
| --- | --- | --- | --- | --- |
|  |  |  | **CHAPSO** | **TX100** |
| **Structural** | ACTC_RAT | Actin, alpha cardiac muscle 1 |  | 3 |
|  | ACTG_RAT | Actin, cytoplasmic 2 | 33 | 132 |
|  | ARP2_RAT | Actin-related protein 2 |  | 4 |
|  | ACTN1_RAT | Alpha-actinin-1 |  | 3 |
|  | AINX_RAT | Alpha-internexin |  | 37 |
|  | BASP_RAT | Brain acid soluble protein 1 | 10 | 28 |
|  | DREB_RAT | Drebrin |  | 13 |
|  | ERC2_RAT | ERC protein 2 | 2 | 6 |
|  | GFAP_RAT | Glial fibrillary acidic protein | 3 | 16 |
|  | LSAMP_RAT | Limbic system-associated membrane protein | 40 | 81 |
|  | MYL6_RAT | Myosin light polypeptide 6 |  | 3 |
|  | MLRA_RAT | Myosin regulatory light chain 2-A, smooth muscle isoform |  | 3 |
|  | MYH10_RAT | Myosin-10 | 1 | 32 |
|  | MYO5A_RAT | Myosin-Va |  | 2 |
|  | NFL_RAT | Neurofilament light polypeptide | 4 | 31 |
|  | NFM_RAT | Neurofilament medium polypeptide |  | 16 |
|  | NEUM_RAT | Neuromodulin | 15 | 45 |
|  | NEGR1_RAT | Neuronal growth regulator 1 | 29 | 67 |
|  | BSN_RAT | Protein bassoon | 10 | 43 |
|  | SPTA2_RAT | Spectrin alpha chain, brain | 6 | 158 |
|  | SPTN2_RAT | Spectrin beta chain, brain 2 |  | 44 |
|  |  |  |  |  |
| **GTPases and Modulators** | RASA3_RAT | Ras GTPase-activating protein 3 | 1 | 5 |
|  | SYGP1_RAT | Ras GTPase-activating protein SynGAP | 1 | 15 |
|  | RAC1_RAT | Ras-related C3 botulinum toxin substrate 1 | 3 | 13 |
|  | RGS7_RAT | Regulator of G-protein signaling 7 | 2 | 18 |
|  | GNA11_RAT | Guanine nucleotide-binding protein alpha-11 subunit | 5 | 3 |
|  | GNA1_RAT | Guanine nucleotide-binding protein G(i), alpha-1 subunit | 37 | 107 |
|  | GNAI2_RAT | Guanine nucleotide-binding protein G(i), alpha-2 subunit | 24 | 45 |
|  | GBB1_RAT | Guanine nucleotide-binding protein G(I)/G(S)/G(T) subunit beta-1 | 26 | 38 |
|  | GBB2_RAT | Guanine nucleotide-binding protein G(I)/G(S)/G(T) subunit beta-2 | 7 | 12 |
|  | GNAI3_RAT | Guanine nucleotide-binding protein G(k) subunit alpha | 4 | 11 |
|  | GNAO_RAT | Guanine nucleotide-binding protein G(o) subunit alpha | 103 | 243 |
|  | GNAQ_RAT | Guanine nucleotide-binding protein G(q) subunit alpha | 13 | 9 |
|  | GNAS2_RAT | Guanine nucleotide-binding protein G(s) subunit alpha isoforms short | 7 | 20 |
|  | GNAZ_RAT | Guanine nucleotide-binding protein G(z) subunit alpha | 15 | 28 |
|  | GBB4_RAT | Guanine nucleotide-binding protein subunit beta-4 |  | 2 |
|  | GBB5_RAT | Guanine nucleotide-binding protein subunit beta-5 | 1 | 3 |
|  |  |  |  |  |
|  |  |  |  |  |

Table 5. Selected protein categories enriched in Triton X100 raft preparations.
